# The association between nutritional status and cognitive impairment in brazilian community-dwelling older adults assessed using a range of anthropometric measures – the Bambui study

**DOI:** 10.1590/S1980-57642013DN74000008

**Published:** 2013

**Authors:** Érico Castro-Costa, Sérgio V. Peixoto, Josélia O.A. Firmo, Elizabeth Uchoa, Maria Fernanda F. Lima-Costa

**Affiliations:** 1 Núcleo de Estudos em Saúde Publica e Envelhecimento, Fundação Oswaldo Cruz/Universidade Federal de Minas Gerais, Belo Horizonte MG, Brazil.; 2 Escola de Enfermagem, Universidade Federal de Minas Gerais, Belo Horizonte MG, Brazil.

**Keywords:** nutritional status, cognitive impairment, anthropometric measures, elderly, population-based

## Abstract

In most studies, body mass index (BMI) has been used as the main measurement of nutritional status. However, BMI does not differentiate between body fat and muscle mass.

**OBJECTIVE:**

To investigate the association between nutritional status and cognitive impairment in a population of Brazilian elderly.

**METHODS:**

Participants (n=1,496) from the Bambuí Cohort Study of Aging were selected based on the results for the two variables nutritional status and cognitive impairment (MMSE score). Gender, age, education, lifestyle, ApoE, chronic diseases, depressive symptoms, current use of hypnotic or sedative medication and functional disability were used as confounding factors for adjusting the logistic regression.

**RESULTS:**

Cognitive impairment was associated with lower BMI (OR: 0.91; CI: 0.86-0.95), waist circumference (OR: 0.97; CI: 0.95-0.99), triceps skinfold thickness (OR: 0.92; CI: 0.89-0.96) among the younger participants (60-69 years), while lower arm muscle circumference (OR: 0.88; CI: 0.80-0.98) and corrected arm muscle area (OR: 0.96; CI: 0.93-0.99) were associated with cognitive impairment among the older participants (70 years and over).

**CONCLUSION:**

There was a difference of association between anthropometric measures and cognitive impairment after stratifying by age group. In the group aged between 60 and 69, cognitive impairment was associated with measures related to fat mass, while in the group aged over 70, cognitive impairment was associated with measures related to muscle mass. This finding suggests that investigation of nutritional status in the elderly using anthropometric measures should not be restricted only to the use of BMI, and should also, differ according to age.

## Abstract (pt)

Na maioria dos estudos o índice de massa corporal (IMC) é usado como a principal medida de avaliação do estudo nutricional. Entretanto, o IMC não apresenta capacidade de diferenciar a gordura corporal da massa muscular.

**OBJETIVO:**

Investigar a associação do estado nutricional e o comprometimento cognitivo na população idosa de Bambuí.

**MÉTODOS:**

Participaram do estudo 1496 idosos que responderam simultaneamente as variáveis do estado nutricional e o comprometimento cognitivo (avaliado através do escore do MMSE). As seguintes variáveis: sexo, idade, educação, estilo de vida, ApoE, doenças crônicas, sintomas depressivos, uso de medicamentos hipnóticos e sedativos e incapacidade funcional foram utilizadas como fatores de confusão na regressão logística multivariada.

**RESULTADOS:**

O comprometimento cognitivo foi associado com os baixos valores de: IMC (OR: 0.91; CI: 0.86-0.95), circunferência da cintura (OR: 0.97; CI: 0.95-0.99), dobra cutânea triciptal (OR: 0.92; CI: 0.89-0.96) entre os idosos mais jovens (60-69 anos). Enquanto que baixos valores da circunferência (OR: 0.88; CI: 0.80-0.98) e da area muscular do braço corrigida (OR: 0.96; CI: 0.93-0.99) foram associados com o comprometimento cognitivo entre os idosos mais velhos (70 anos ou mais).

**CONCLUSÃO:**

Existe uma diferença entre a associação das medidas antropométricas e o comprometimento cognitivo após a estratificação por idade. Nos participantes entre 60 a 69 anos, o comprometimento cognitivo foi associado a medidas relacionadas com o tecido adiposo enquanto que no grupo com 70 anos ou mais, o comprometimento cognitivo foi associado a medidas relacionadas com a massa muscular. Esses achados sugerem que a investigação do estado nutricional dos idosos não se deve restringir somente ao IMC, sendo necessário variações devido a idade.

## INTRODUCTION

Malnutrition is a common problem among older adults,^1^ leading to severe complications such as weakening of the immune system, muscle loss, loss of independence and increased mortality.^2^ In addition, malnutrition in the elderly is associated with several geriatric syndromes including cognitive deficit,^3,4^ which represents one of the most disabling health conditions in the elderly.^5^

Recent studies have confirmed a higher prevalence of malnutrition in older adults with different types of cognitive impairment (dementia, mild cognitive impairment).^5-7^ However, in most of these studies body mass index (BMI) was used as the main measurement of nutritional status. It is well known that BMI can be misleading in terms of a person's body fat percentage as the index solely takes account of net weight and height of an individual and does not consider the distribution of muscle and bone mass. It also fails to differentiate between body fat and muscle mass.^8^

In older people, there tends to be an increase in body weight and fat tissue and a decrease in muscle mass. Therefore, the use of BMI as a nutritional measurement may underestimate the body fat percentage in those with less lean body mass, such as elderly individuals. In order to overcome this limitation, the Nutrition Screening Initiative (NSI)^9^ and Lipschitz^10^ proposed a different BMI cut-off as suggested by the World Health Organization.^11^ However, there is no universal agreement on which BMI cut-off point should be used and how to define malnutrition using BMI values.^12^ Also, Guo et al., 1999^13^ hold that BMI is not appropriate for assessing levels of nutritional status in the elderly because it does not provide information about distribution of fat in different parts of the body.

In addition, recent evidence has indicated that alterations in body composition are apparent in the earliest clinical stage of Alzheimer's disease and have furthered these findings in suggesting that Alzheimer's disease-related alterations in body composition may be predominantly due to loss of lean mass (i.e. sarcopenia).^14^ This is consistent with at least one large epidemiological study that found an association between cognitive impairment and reduced muscle mass in women without dementia.^15^ These findings suggested that lean mass may be a more sensitive measure to relate body composition to cognitive outcomes and to dementia than measures of adiposity.

Although there are other anthropometric measures that may be more reliable and easier to perform, they have only been used in a few studies.^16,17^ Taking into account the changes in body composition during aging and the limitations of BMI for assessing levels of nutritional status in the elderly, the aim of this study was to investigate the association between nutritional status and cognitive impairment, supplementing data on BMI with other anthropometric measures in a well-defined population of Brazilian elderly with low-educational levels.

## METHODS

**The Bambuí Health and Ageing Study (BHAS).** The BHAS is an ongoing population-based prospective cohort study of older adults carried out in Bambuí (15,000 inhabitants), a city in the State of Minas Gerais, Southeast Brazil. The data presented in this paper were collected at the baseline examination. A detailed description of the design and methods of the study has been published previously.^18,19^

**Study population.** All residents aged 60 years and over were eligible to take part in the BHAS baseline and were identified through a complete census of the city, carried out by the research team between November and December 1996. Of 1,742 residents identified who were aged 60 years and over, 1,606 (92.2%) were interviewed and received comprehensive health status examinations. Those interviewed and examined were similar to the total population in Bambuí aged 60 years and over with respect to all sociodemographic characteristics considered, including age, gender, number of residents in the household, marital status and educational level.^18,19^

**Measures.** *Cognition assessment – *The BCSA includes a standard Brazilian version of the MMSE,^20^ which was completed annually. In the Brazilian validation study, some questions were modified according to their relevance to the target population. In the orientation section by Folstein et al.,^21^ questions about the season of the year, building and floor were replaced by period of the day, room and address in the Brazilian version. In the registration and recall section, the words used were "cat", "tree" and "guitar". In the attention and calculation section, serial fives replaced serial sevens and "Maria," which is more commonly used in the Brazilian culture, replaced spelling "world" backwards. Despite potential questions that could be raised concerning the cross-cultural equivalence of the MMSE, a previous study in this population provided some support for this equivalence, suggesting that most of the items and underlying constructs remain meaningful, even after modification and translation, in a low-education population with a lower overall distribution of scores.^22^ In the absence of comparable cut-off points, percentile distribution of MMSE scores is more appropriate for population-based studies of the elderly with low schooling level. In this study, cognitive impairment was defined using the cut-off point of 21/22, which corresponded to the lower quartile of the MMSE score distribution.^23^

*Nutritional assessment – *Various methods were used for assessing nutrition. In the BHAS, traditional anthropometric measures were used to assess nutritional status because they were easy to perform and frequently applied in epidemiological studies.^4,16^ For the present study, anthropometric measures were divided into principal measures (weight, height, waist circumference – WC, triceps skinfold thickness -TST, mid-arm circumference - MAC) and derived measures (body mass index – BMI, arm muscle circumference – AMC and corrected arm muscle area – CAMA).

All principal anthropometric measurements were taken three times by a trained team; the mean was considered for the purposes of analysis. Waist circumference (WC) was measured with a tape measure placed midway between the lower rib margin (costal margin) and the superior anterior iliac spine (iliac crest) to the nearest 0.5 cm, in a standing position. Triceps skinfold thickness (TST) was taken with the person standing upright with their arms hanging down loosely. The skin fold was pulled away from the muscle and measured with calipers, taking a reading 4 seconds after the calipers had been released. The point of measurement was half way between the olecranon process of the ulna and the acromion process of the scapula. Mid-arm circumference was the circumference of the left upper arm, measured at the mid-point between the tip of the shoulder and the tip of the elbow (olecranon process and the acromion).

Regarding derived measures, weight and height measurements were used to calculate BMI (weight/height^2^). The arm muscle circumference (AMC) is derived from the mid-arm circumference (MAC) and the triceps skinfold thickness (TSF) calculated by accounting for the thickness of the subcutaneous fat that surrounds the muscle, using the following formula, with the mid-arm circumference (MAC) and triceps skinfold thickness (TSF) values.^24^

$$\mathrm{AMC}\left(\mathrm{cm}\right)=\left[\mathrm{MAC}\left(\mathrm{cm}\right)-\left(\pi \mathrm{xTSF}\left(\mathrm{cm}\right)\right)\right]$$

The *corrected* arm muscle area (CAMA) is derived from the arm muscle circumference (AMC) using the following two formulae, with the MAC and TSF values in the equation developed by Heymsfield et al., 1982.^25^

$$\begin{array}{c} \mathrm{For}\;\mathrm{men}\\ \frac{\mathrm{CAMA}\left({\mathrm{cm}}^{2}\right)={\left[\mathrm{MAC}\left(\mathrm{cm}\right)-\left(\pi \mathrm{xTSF}\left(\mathrm{cm}\right)\right)\right]}^{2}-10}{4\pi }\\ \mathrm{For}\;\mathrm{womem}\\ \frac{\mathrm{CAMA}\left({\mathrm{cm}}^{2}\right)={\left[\mathrm{MAC}\left(\mathrm{cm}\right)-\left(\pi \mathrm{xTSF}\left(\mathrm{cm}\right)\right)\right]}^{2}-6.5}{4\pi } \end{array}$$

**Covariates.** Information on gender, age and schooling level (number of complete years of schooling) was obtained in the baseline BHAS interview. Participants were asked about the following lifestyle characteristics: current smoking and alcohol consumption. Current smokers were defined as those who had smoked at least 100 cigarettes during their lifetime and were still smoking. Alcohol consumption was defined as any consumption of alcohol in the last 12 months. Genomic DNA for ApoE genotyping was extracted from blood samples using the Wizard^®^ Genomic DNA Purification System (Promega, Madison, WI, USA). Consistent with the seventh Joint National Committee criteria, hypertension was defined as systolic blood pressure greater than or equal to 140 mmHg, and/or diastolic blood pressure greater than or equal to 90 mmHg and/or the use of antihypertensive drugs.^26^ Diabetes mellitus was defined as fasting blood glucose greater than or equal to 126 mg/dl and/or current treatment for diabetes, following the 2003 American Diabetes Association updated criteria.^27^ The presence of depressive symptoms was defined as a score of over 4 on the 12-item General Health Questionnaire (GHQ-12).^28^ The GHQ-12 was originally developed for assessing common mental disorder, however, this Brazilian validated version of the GHQ-12 has been found to be equivalent to the 30-item Geriatric Depression Scale (GDS-30) for detecting depression symptoms in this elderly sample.^28^ Information about the use of hypnotic or sedative medication was collected using the sleep questionnaire previously described.^29^ Current use of hypnotic or sedative medication was defined as any number of pills taken in the last month. Functional disability was defined as the inability to perform at least one of five activities of daily living: dressing, eating, walking around inside the house, bathing and maintaining urinary continence.^30^

The interviews were carried out by community members with at least 11 years of schooling selected by the research team. The interviewers were trained by a psychiatrist with extensive experience in different countries and cultures.^18,19^ Nutritional assessment was also performed by community members carefully trained by a nutritionist. The BHAS was approved by the Research Ethics Committee of the Oswaldo Cruz Foundation.

**Statistical analysis.** Unadjusted associations of cognitive impairment with sociodemographic factors, apoeE allele 4, lifestyle characteristics, current use of psychoactive medication and anthropometric measures were evaluated using Pearson χ^2^ tests for linear trend or Student *t-*tests. Categorical variables were: gender, schooling level, ApoE allele 4, smoking, alcohol consumption, hypertension, diabetes, depression symptoms, use of psychoactive medication, and functional disability. Age and all anthropometric measures were treated as continuous variables. In addition, the Student *t-*test was used to determine if there was a significant difference between the mean (SD) of all anthropometric measures from participants with or without cognitive impairment in the different age groups (60-69 years and 70 years and over). Finally, an adjusted logistic regression analysis was performed to confirm the association between malnutrition evaluated using different anthropometric measures and cognitive impairment. The STATA software Package (version 10.1, College Station, TX, USA) was used for all analyses.^31^

## RESULTS

Of the 1,606 cohort members, those included in the present analysis were the 1,496 baseline participants for whom both cognitive and nutritional status were determined (110 were excluded for refusals to perform blood tests). Most subjects were women and the mean age of participants was 68.9 years. Thirty-five percent had more than 4 years of education. Compared against this same age group in the general population, the participants in this study had more years of schooling (df: 2; χ^2^=23.9; p<0.001).

The prevalence of cognitive impairment was 19.6%, the overall mean (SD) of BMI was 25.1 (5.0), while 23.5 (4.9) was the mean (SD) among those with cognitive impairment. Means of others anthropometric parameters were also lower in participants with cognitive impairment than those without cognitive impairment. Further characteristics of the study population including details of cognitive impairment are shown in Table 1.

**Table 1:** Characteristics of the study participants, by cognitive impairment

Characteristics		Cognitive impairment			
		Total n=1496	Positive (MMSE <22) n=293	Negative (MMSE ≥22) n=1203	p-value
Age, years		68.9 (7.1)	71.1 (8.1)	68.4 (6.8)	**<0.0001**
Females, %		61.4	53.1	63.4	**<0.0001**
Schooling level (≥4 years), %		35.9	9.8	42.3	**<0.0001**
APOE Allele 4, %	Absent	74.9	72.2	75.4	
	Heterozygosis	23.3	23.3	23.3	
	Homozygosis	1.8	4.5	1.3	**0.0050**
Currently smoking, %		18.1	25.5	16.3	**<0.0001**
Alcohol consumption, %		18.3	19.7	17.9	0.4790
Hypertension, %		62.0	67.6	60.8	**0.0410**
Diabetes, %		15.1	12.9	15.6	0.2910
Depression symptoms (GHQ-12≥5), %		38.5	52.0	35.1	**<0.0001**
Current use of psychoactive medication, %		21.8	18.0	22.7	0.0840
Rate of functional disability (Katz), %		11.1	17.1	9.7	**<0.0001**
Anthropometric measures	BMI (kg/m^2^)	25.1 (5.0)	23.5 (4.9)	25.5 (4.9)	**<0.0001**
	WC (cm)	91.3 (11.3)	88.1 (12.4)	92.0 (11.0)	**<0.0001**
	TST (mm)	17.4 (8.8)	14.1 (7.6)	18.1 (8.9)	**<0.0001**
	MAC (cm)	28.1 (4.0)	26.5 (4.0)	28.5 (3.9)	**<0.0001**
	AMC (cm)	22.7 (3.0)	22.1 (2.9)	22.9 (3.1)	**0.0011**
	CAMA (cm^2^)	34.0 (10.7)	31.6 (9.9)	34.6 (10.8)	**<0.0001**

BMI: body mass index; WC: waist circumference; TST: triceps skinfold thickness; MAC: mid arm circumference; AMC: arm muscle circumference; CAMA: corrected arm muscle area. Values for age, BMI, WC, TST, MAC, AMC, CAMA, are expressed as mean (SD). p-value: Student's t-test, Person's c2 test for differences between means and frequencies, respectively.

Table 2 shows the anthropometric profile of participants by age and cognitive impairment. All anthropometric parameter means were smaller in older participants with cognitive impairment, while only means of BMI, waist circumference, triceps skinfold thickness and mid-arm circumference were smaller in younger participants with cognitive impairment.

**Table 2:** Baseline characteristics according to age and cognitive impairment.

Variables		60-69 years					70 years or older			
		MMSE<22	MMSE ≥22	Total	p-value		MMSE<22	MMSE ≥22	Total	p-value
**Anthropometric profile**	BMI (kg/m^2^)	23.6 (4.5)	25.9 (5.0)	25.6 (4.9)	<0.0001		23.3 (5.3)	24.9 (4.7)	24.6 (4.9)	0.0013
	WC (cm)	88.1 (12.4)	92.0 (11.0)	91.3 (11.3)	<0.0001		87.9 (11.6)	91.9 (11.0)	91.3 (11.2)	0.0001
	TST (mm)	14.0 (7.3)	19.0 (9.4)	18.2 (9.3)	<0.0001		14.3 (8.1)	16.7 (7.7)	16.2 (7.8)	0.0019
	MAC (cm)	27.3 (3.7)	29.1 (3.9)	28.8 (3.9)	<0.0001		25.7 (4.2)	27.5 (3.9)	27.1 (4.0)	<0.0001
	AMC (cm)	22.8 (3.0)	23.2 (3.1)	23.1 (3.1)	0.2115		21.4 (2.7)	22.3 (2.9)	22.1 (2.8)	0.0014
	CAMA (cm^2^)	33.8 (10.3)	35.8 (10.9)	35.4 (10.8)	0.0302		29.1 (8.9)	32.6 (10.4)	31.8 (10.2)	0.0004

BMI: body mass index; WC: waist circumference; triceps skinfold thickness; MAC: mid-arm circumference; AMC: arm muscle circumference; CAMA: corrected arm muscle area. Values for BMI, WC, TST, MAC, AMC, CAMA are expressed as mean (SD). p-value: student's t-test for differences between medians.

Table 3 shows adjusted OR for many potential confounders for the association between MMSE score and different anthropometric measures by age. Cognitive impairment was associated with lower BMI (OR: 0.91; CI: 0.86-0.95), waist circumference (OR: 0.97; CI: 0.95-0.99), TST (OR: 0.92; CI: 0.89-0.96) among the younger participants, while lower arm muscle circumference (OR: 0.88; CI: 0.80-0.98) and corrected arm muscle area (OR: 0.96; CI: 0.93-0.99) were associated with cognitive impairment among the older participants. Mid-arm circumference was associated with cognitive impairment in both groups.

**Table 3:** Results of analyses of association between cognitive impairment, anthropometric and biochemistry profile by age.

Variables		OR (95% CI)*		
		60-69 years	70 years or over	Total
Anthropometric profile	BMI (kg/m^2^)	0.91 (0.86-0.95)	0.95 (0.90-1.01)	0.92 (0.89-0.96)
	WC (cm)	0.97 (0.95-0.99)	0.98 (0.96-1.01)	0.97 (0.96-0.99)
	TST (mm)	0.92 (0.89-0.96)	0.97 (0.94-1.01)	0.94 (0.92-0.97)
	MAC (cm)	0.89 (0.84-0.95)	0.92 (0.86-0.98)	0.90 (0.86-0.94)
	AMC (cm)	0.94 (0.87-1.02)	0.88 (0.80-0.98)	0.92 (0.87-0.98)
	CAMA (cm^2^)	0.98 (0.96-1.00)	0.96 (0.93-0.99)	0.97 (0.96-0.99)

BMI: body mass index; WC: waist circumference, triceps skinfold thickness; MAC: mid-arm circumference; AMC: arm muscle circumference; CAMA: corrected arm muscle area. OR (95% CI) estimated by logistic regression. All results were adjusted for age, gender, schooling level, ApoE, currently smoking, alcohol consumption, hypertension, diabetes, depression symptoms (GHQ≥5), current use of psychoactive drugs (hypnotics, benzodiazepines), rate of functional disability. Bold p-value <0.05

## DISCUSSION

This paper describes, to the best of our knowledge, is the first investigation in Brazil on the association between nutritional status (using various anthropometric measures) and cognitive impairment in community-dwelling older adults, taking into account a range of potential confounding factors. In Bambuí, different anthropometric measures were associated with cognitive impairment in different age groups. BMI and waist circumference were associated with cognitive impairment in young elderly while arm muscle circumference and corrected arm muscle area were associated in older elderly – and these associations persisted after controlling for sociodemographic status, ApoE, lifestyle, chronic diseases, depression, use of psychoactive drugs and functional disability.

The study has a number of strengths including the use of a community-based population, its large sample size, high response rate, use of other anthropometric measures, instead of only BMI, for assessing nutritional status, the intensive training of the field and laboratory teams and the adjustment for a range of potential cofounders including; chronic diseases, current use of psychoactive medication and functional disability. Also, the use of anthropometric measures as continuous variables helps to avoid measurement inaccuracies, which can occur when using established cut-off points.^32^

The study does have some limitations. Firstly, due to the challenging logistics, we did not use other measures such as bioelectrical impedance spectroscopy. However, although the use of these measures is desirable, their incorporation into community epidemiological studies poses substantial logistic challenges and the anthropometric measures remain the most widely used instruments in such studies.^4^ Secondly, on the basis of cost, we could not use measures such as dual-energy X-ray absorptiometry (DEXA), hydrodensitometry and whole-body potassium which are accurate methods often cited in other studies,^33^ In addition to the question of cost, these measurements require a high level of cooperation from the individual, which may be complicated to achieve in an older population with severe physical and cognitive impairments.^34^ Finally, populations with low schooling levels tend to exhibit worse performance on the MMSE. However, several strategies have been proposed in order to minimize the effects of schooling level in the interpretation of MMSE score. In Brazil, the most common approach has been the use of different cut-off points according to schooling level,^35^ although some evidence has failed to support this approach.^36^ To overcome this inconsistency, we used a cut-off point based on the distribution of the MMSE scores in the study population.^23^

Recently, many studies have demonstrated the association between nutritional status and cognitive impairment among hospitalized old people and those living in elderly care homes.^7,37^ However, studies investigating the association between nutritional status and cognitive impairment in older community-dwelling populations are scarce.^1,4^ Lee et al. (2009)^1^ investigated nutritional status in a sample of 2934 non-institutionalized older individuals aged 60 years or older from an urban community of China and found an association between nutritional risk and cognitive impairment even after adjusting for demographics and depression. Also, Fagerstrom et al. (2011),^4^ investigating data on nutritional characteristics in 1,402 people aged 60 years and older who lived in regular or special housing in a municipality in south-eastern Sweden, demonstrated that malnutrition was associated with cognitive impairment in both those living in special and regular housing, but was more pronounced in the former group. However, both these previous reports did not investigate the association of other anthropometric measures with cognitive impairment.

In the study described here, nutritional status was assessed not only for BMI but also complemented with waist circumference, triceps skinfold thickness, mid-arm circumference, arm muscle circumference and corrected arm muscle area. All anthropometric measures were associated with cognitive impairment, and had very similar odds ratios in separate models for the whole population. However, final models stratified according to age (from 60 to 69 years and 70 years and older), where each anthropometric measure was entered with all other covariates, indicated differences in the association of those variables and cognitive impairment. Measures related to fat mass (waist circumference and triceps skinfold thickness) were associated with cognitive impairment in younger old people, while measures related only to muscle mass (arm muscle circumference, corrected arm muscle area) were associated with older elderly. On the other hand, mid-arm circumference remained associated with cognitive impairment in both age groups.

These findings might be explained by patterns of change in body composition over time in older adults, where the percentage of fat mass increases initially, then decreases^38^ and by skeletal muscle mass decline, especially in adults older than 70 years.^39^ On the other hand, our results with BMI and mid-arm circumference may have been attributed to the estimation of these measures. BMI includes height, which in elderly persons decreases with age, and thus introduces inaccuracy and an overestimation of the body fat measure, whereas mid-arm circumference reflects muscle mass as well as fat mass.

In conclusion, the present study shows that there is a relationship between nutritional status measured by different anthropometric methods and cognitive impairment among elderly living in the community. The major finding in this population of older people from Bambuí was the difference of association between anthropometric measures and cognitive impairment after stratifying by age group. This finding suggests that the investigation of nutritional status in the elderly using anthropometric measures should not be restricted only to the use of BMI and should also differ according to age. Further studies are necessary to better understand this complex relationship between nutritional status and cognitive impairment.
